# Systemic delivery of oncolytic herpes virus using CAR-T cells enhances targeting of antitumor immuno-virotherapy

**DOI:** 10.1007/s00262-024-03757-8

**Published:** 2024-07-02

**Authors:** Zongliang Zhang, Nian Yang, Long Xu, Huaqing Lu, Yongdong Chen, Zeng Wang, Qizhong Lu, Kunhong Zhong, Zhixiong Zhu, Guoqing Wang, Hexian Li, Meijun Zheng, Liangxue Zhou, Aiping Tong

**Affiliations:** 1 State Key Laboratory of Biotherapy and Cancer Center, Research Unit of Gene and Immunotherapy, Chinese Academy of Medical Sciences, Collaborative Innovation Center of Biotherapy, West China Hospital, Sichuan University, Chengdu, 610041 Sichuan Province China; 2 Department of Ophthalmology, West China Hospital, Sichuan University, West China Medical School, Chengdu, 610041 Sichuan China; 3 Department of Otolaryngology, Head and Neck Surgery, West China Hospital, West China Medical School, Sichuan University, Chengdu, 610041 Sichuan China; 4 Department of Neurosurgery, West China Hospital, West China Medical School, Sichuan University, Chengdu, 610041 Sichuan China; 5 Department of Neurosurgery, Fifth People’s Hospital of Ningxia Hui Autonomous Region, Shizuishan, 753000 Ningxia China; 6 Department of Neurosurgery, Mianyang Central Hospital, Mianyang, 621000 Sichuan China; 7 Frontiers Medical Center, Tianfu Jincheng Laboratory, Chengdu, 610212 China

**Keywords:** CAR-T, B7-H3, HSV, Systemic delivery, Oncolytic virotherapy

## Abstract

Recent studies have indicated that combining oncolytic viruses with CAR-T cells in therapy has shown superior anti-tumor effects, representing a promising approach. Nonetheless, the localized delivery method of intratumoral injection poses challenges for treating metastatic tumors or distal tumors that are difficult to reach. To address this obstacle, we employed HSV-1-infected CAR-T cells, which systemically delivery HSV into solid tumors. The biological function of CAR-T cells remained intact after loading them with HSV for a period of three days. In both immunocompromised and immunocompetent GBM orthotopic mouse models, B7-H3 CAR-T cells effectively delivered HSV to tumor lesions, resulting in enhanced T-cell infiltration and significantly prolonged survival in mice. We also employed a bilateral subcutaneous tumor model and observed that the group receiving intratumoral virus injection exhibited a significant reduction in tumor volume on the injected side, while the group receiving intravenous infusion of CAR-T cells carrying HSV displayed suppressed tumor growth on both sides. Hence, CAR-T^HSV^ cells offer notable advantages in the systemic delivery of HSV to distant tumors. In conclusion, our findings emphasize the potential of CAR-T cells as carriers for HSV, presenting significant advantages for oncolytic virotherapy targeting distant tumors.

**Supplementary Information:**

The online version contains supplementary material available at 10.1007/s00262-024-03757-8.

## Introduction

In recent years, CAR-T cell therapy has displayed promising outcomes in treating hematologic malignancies. The U.S. Food and Drug Administration (FDA) has granted approval for CAR-T cell therapy to be used in lymphoma and acute lymphoblastic leukemia treatments [1, 2]. However, the application of CAR-T cell therapy in solid tumor treatment encounters challenges due to the immunosuppressive conditions present within the tumor microenvironment (TME) [3, 4]. These challenges include limited transport abilities of CAR-T cells, difficulties in identifying ideal tumor antigens, and the diminished persistence and proliferation of CAR-T cells.

Unlike dispersed blood tumor cells, solid tumors often form compact masses surrounded by abundant tumor-associated fibroblasts (CAFs) and blood vessels, thus creating natural physical barriers [5, 6]. Moreover, certain solid tumors have the capability to inhibit the secretion of specific chemokines. The interaction between chemokines and their receptors facilitates the migration of T cells into the tumor microenvironment [7]. However, CAR-T cells lack surface receptors that correspond to the chemokines secreted by solid tumors, resulting in the limited ability of CAR-T cells to home in on tumor sites. Studies have revealed that the TME is characterized by low pH, hypoxia, high osmolarity, and the presence of immunosuppressive mechanisms, all of which are highly detrimental to the survival and immunological effectiveness of T cells [8]. Immunosuppressive cells, including regulatory T cells (Tregs), myeloid-derived suppressor cells (MDSCs), and M2 macrophages, are present within the TME of solid tumors. These immunosuppressive cells release cytokines such as transforming growth factor-beta (TGFβ) [9, 10] and interleukin-10 (IL-10) [11, 12], which diminish the anti-tumor effects of CAR-T cells upon infusion. Consequently, the development of novel strategies to overcome the complex immunosuppressive microenvironment in solid tumors is a pressing matter.

Oncolytic viruses have emerged as a promising therapeutic strategy in the field of cancer treatment. These genetically engineered viruses possess desirable tumor-selective properties, immunogenicity, and the capacity to deliver targeted transgenes to tumors [13, 14]. Currently, oncolytic herpes viruses (oHSV) have demonstrated the ability to selectively replicate within and eliminate tumor cells in tumors that lack the interferon signaling pathway activity, which encompasses more than 90% of the total tumor cell population [15]. T-Vec, the first oncolytic virus to be approved by the FDA in the US, is a genetically modified oHSV that expresses granulocyte–macrophage colony-stimulating factor (GM-CSF) and is employed for melanoma treatment [16]. G47Δ, by incorporating three mutations into the HSV genome, one of which involves the knockout of the ICP47 gene found in the second-generation oncolytic virus (G207), shows enhanced selective replication within tumor cells [17, 18]. This leads to tumor cell lysis and activation of the human immune system, ultimately resulting in the destruction of tumor cells. Preclinical studies have explored the combination of oncolytic HSV with CAR-T cell therapy for solid tumors [19, 20]. The local intratumor injection of the oncolytic virus has the potential to augment the trafficking of CAR-T cells into the virus-injected tumors, modulate the local immunosuppressive environment, and enhance the effector function of CAR-T cells. Additionally, there have been endeavors to engineer oncolytic viruses with payloads such as cytokines, chemokines, and immune checkpoint inhibitors [21]. This approach aims to convert “cold” tumors, which lack immune cell infiltration, into “hot” tumors that exhibit improved responsiveness to CAR-T cell therapy.

The rise in popularity of oncolytic herpes simplex viruses stems from their capacity to elicit immune-stimulatory effects within the localized tumor microenvironment. Unfortunately, the conventional intravenous infusion of oncolytic viruses has thus far failed to achieve adequate viral enrichment within tumor sites [22, 23]. A significant hurdle in the extensive utilization of these viral agents for treating metastatic conditions persists in the challenge of delivering them systemically to well-established tumors that are not directly accessible for injection. This difficulty arises, in part, from the neutralization of viruses, nonspecific binding of virus particles to various host cell types, and the active retention of particles. Additionally, there is the additional issue of localizing viruses exclusively to tumors and facilitating their passage from the bloodstream into surrounding tissues. Previous investigations have explored alternative strategies to enhance the systemic delivery of oncolytic viruses (OVs) to tumors, including the utilization of T cell-mediated viral delivery [24–27]. A recent study employed CAR-T and TCR-T cells as vehicles for delivering the myxoma oncolytic virus (MYXV), with the T cells being pre-infected ex vivo using a spin infection protocol [28]. Tumor-specific CAR-T and TCR-T cells effectively transported MYXV into the corresponding tumor cells in a manner specific to the associated antigen, while sparing normal cells. In line with this, we employed B7-H3 CAR-T cells as carriers for an oHSV, derived from our laboratory, intended to exert an antitumor effect upon delivery into tumor cells. B7-H3, belonging to the B7 ligand family, exhibits highly expressed in human malignancies, while exhibiting restricted expression within normal tissues [29, 30]. Several CAR-T cells designed to specifically target B7-H3 have been meticulously formulated and have undergone comprehensive evaluation in clinical trials [29–32]. The modified oHSV consists of three mutations, including deletions in the antigen processing inhibition gene (ICP47), two copies of the neurovirulent factor (ICP34.5), and the ribonucleotide reductase gene (ICP6), which collectively enhance the safety and effectiveness of the oHSV. To the best of our knowledge, this is the inaugural instance wherein a systemically delivered oHSV has been facilitated by CAR-T cells targeting solid tumors. Unexpectedly, both in vitro and in vivo studies demonstrated the efficient delivery of the oHSV by B7-H3 CAR-T cells into the corresponding tumor cells, leading to tumor suppression.

## Materials and methods

### Infecting of HSV to *CAR*-T cells

To assess the infectivity of HSV on the CAR-T cells, HSV^dko^-GFP was introduced to the CAR-T cells at a multiplicity of infection (MOI) of 4:1 and incubated with the cells for a continuous 5-day culture period. The presence of CAR-T^HSV^ cells was identified through immunofluorescence and flow cytometry analyses. The viability of B7-H3 CAR-T cells was determined by employing the trypan blue exclusion test to obtain a relative count of viable cells.

### Tumor models and treatments

In order to establish the GBM orthotopic mice model, NCG mice were appropriately anesthetized and underwent a stereotactic procedure whereby 1 × 10^5^ U87 cells were skillfully injected into the right frontal lobe of the brain. The precise coordinates for the injection were determined as being 2 mm lateral and 1 mm anterior to bregma, at a depth of 3 mm. After a 7-day period following tumor implantation, the mice were intravenously administered either B7-H3 CAR-T cells or B7-H3 CAR-T^HSV−Luci^ cells. Simultaneously, intratumoral injections of either PBS or 2 × 10^5^ PFU HSV-Luci were performed. Similarly, C57BL/6 mice were subjected to stereotactic injection of 1 × 10^5^ GL261-hB7-H3 cells into the same specified brain region mentioned previously. Consistent with the previous experimental protocol, after the tumors had been implanted for 7 days, the mice received intravenous injections of either B7-H3 mCAR-T cells or B7-H3 mCAR-T^HSV−Luci^ cells. Additionally, separate intratumoral injections were administered, consisting of 2 × 10^5^ PFU HSV-Luci or PBS.

In the subcutaneous mice model, NCG mice were subjected to subcutaneous injections of 5 × 10^6^ U87 cells. When the tumors reached approximately 80 mm^3^ on day 15, intravenous injections of either B7-H3 CAR-T cells or B7-H3 CAR-T^HSV−Luci^ cells were administered. In certain experiments, only tumors on the left flanks received intratumoral injections of either PBS or HSV-Luci; tumors on the right flanks did not receive such injections. After a 3-day period following the administration of treatment, the mice were anesthetized with isoflurane and intraperitoneally injected with 100 μ L of 15 mg/mL D-Luciferin (Beyotime). This allowed for imaging using the IVIS imaging system (Caliper Life Sciences) to obtain luciferase-based in vivo images, enabling the evaluation of HSV-Luci infection within the tumors. Furthermore, the mice were regularly monitored in terms of tumor progression and weight. At the specified time points or at the experimental endpoint, the mice were euthanized.

### Statistical analyses

Statistical analyses were conducted using Prism 9 (GraphPad). Comparisons between two independent conditions were assessed using the unpaired two-tailed Student’s t-test, while comparisons involving multiple independent conditions were analyzed using one-way ANOVA. Survival data were analyzed using the Kaplan–Meier method, and the log-rank test was employed for comparisons. The results are expressed as mean ± standard deviation (SD), unless otherwise specified. A *P* value of 0.05 or less was considered statistically significant.

## Results

### HSV infected *CAR*-T cells

**Fig. 1:**
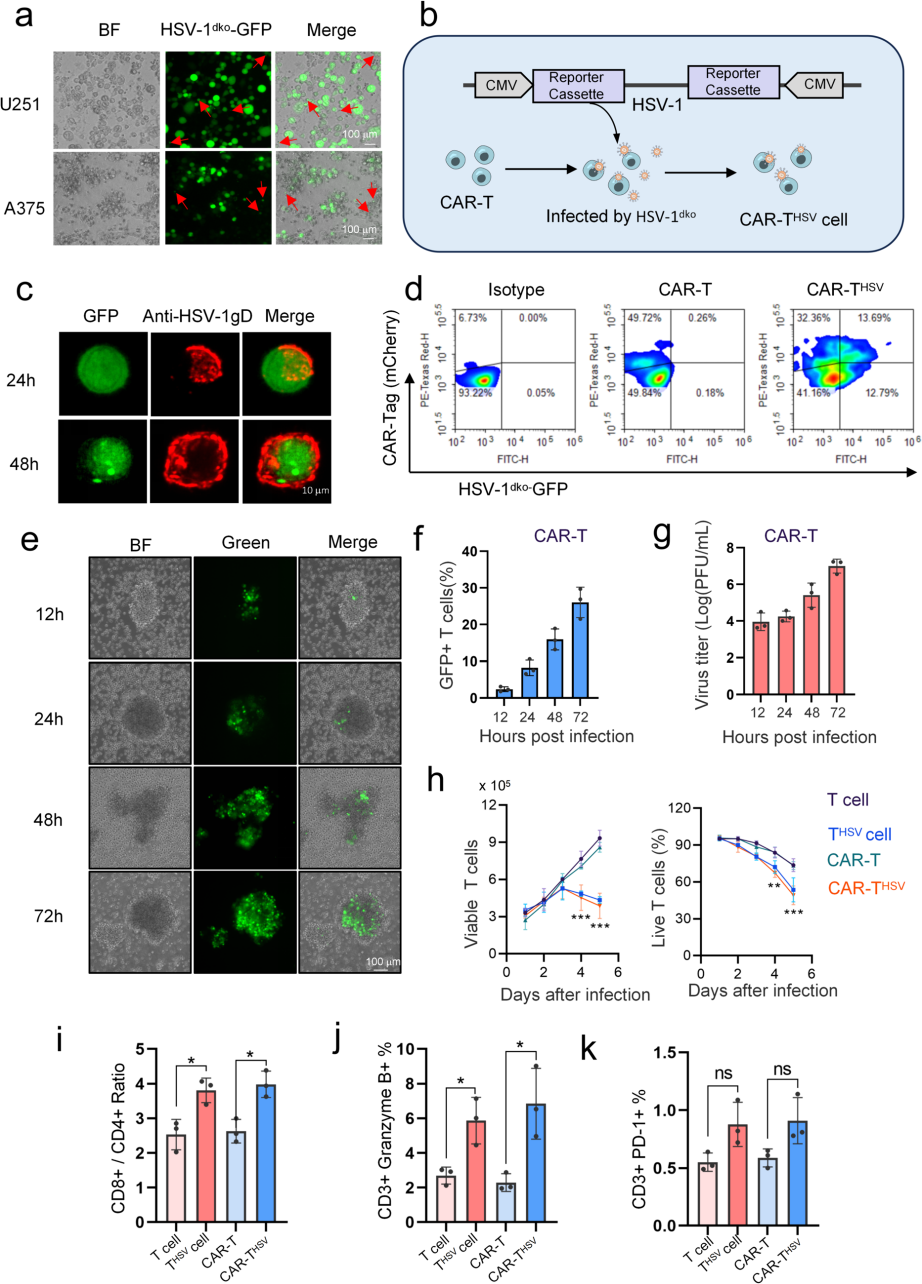
Delivery of HSV by CAR-T cells. **a** After infecting HSV-1^dko^-GFP 24 h, A375 and U251 tumor cells were cocultured with B7-H3 CAR-T cells. It was observed that B7-H3 CAR-T cells exhibited susceptibility to infection by the HSV-1^dko^-GFP virus (red arrow). **b** The schematic illustrates the preparation of CAR-T^HSV^ cells. **c** At 24 and 48 h post-viral infection, immunofluorescence staining was conducted on B7-H3 CAR-T cells using anti-HSV-1gD antibodies, and the resulting images were analyzed using a scale bar of 10 µm. **d** The infection rate at 48 h was assessed through the utilization of flow cytometry. Over the course of time, the addition of HSV-1^dko^-GFP to T cells or CAR-T cells demonstrated an enhancement in the rate of HSV-1^dko^-GFP infection (e and f), and this led to an escalation in viral replication (**g**). The yield of viable uninfected (PBS), T^HSV^ cells and CAR-T^HSV^ cells were measured by trypan blue exclusion test, as well as the percentages of live T^HSV^ cells and CAR-T^HSV^ cells (**h**). n = 3/group. Values are presented as mean ± SD. **P < 0.01; ***P < 0.001, two-way ANOVA with post hoc Holm-Sidak test. At 24 h post-viral infection, the CD8+/CD4+ ratio (**i**), cytokine secretion (**j**), and expression levels of PD-1 (**k**) were conducted. BF: brightfield, Green: oncolytic HSV-1 (GFP). n = 3/group. Values are presented as mean ± SD. *P < 0.05; one-way ANOVA with Tukey test

In our previous investigations, B7-H3 CAR-T cells demonstrated notable antitumor efficacy when co-cultured with various solid tumors in vitro. Oncolytic herpes simplex virus (oHSV) has emerged as a promising and safe oncolytic virus capable of selectively infecting and eliminating diverse tumor cell types. It is evident that combining oHSV with B7-H3 CAR-T cells holds potential as a synergistic therapeutic approach. In our current study, we sought to explore the possibility of utilizing CAR-T cells as carriers for delivering oHSV, presenting a novel approach distinct from traditional combination strategies. An accidental discovery was made during the co-culturing of B7-H3 CAR-T cells with U251 or A375 tumor cells that had been infected with HSV-1^dko^-GFP for 24 h. It was observed that B7-H3 CAR-T cells could be infected with the HSV-1^dko^-GFP virus (Fig. 1a). To assess the level of infection, GFP-expressing reporter HSV-1^dko^ was directly added to CAR-T cells at a multiplicity of infection (MOI) of 4:1 and incubated for 48 h. The extent of infection was evaluated through GFP and anti-HSV-1gD (red) imaging (Fig. 1b, c). Furthermore, flow cytometry was employed to detect the rate of HSV-1^dko^ infection in CAR-T cells (Fig. 1d). Simultaneously, we examined the infection status of the HSV virus (MOI = 1) in a co-culture system of U87 cells and T cells at a 1:1 ratio. The results indicate that when the HSV is introduced into a co-culture of tumor cells and T cells, it preferentially infects U87 cells without conspicuously infecting the T cells (Fig. S1a).

Over time, as the HSV-1^dko^-GFP was added to CAR-T cells, the rate of HSV-1^dko^ infection showed improvement (Fig. 1e, f), resulting in increased viral replication (Fig. 1g). In an attempt to evaluate the progressive killing capacity of CAR-T^HSV^ in vitro, CAR-T cells were loaded with viruses for durations of 3, 5, and 7 days, followed by co-culturing with tumor cells (E:T = 4:1). Subsequently, the expression levels of Granzyme B secreted by alive T cells were measured to assess the cytotoxic potential of CAR-T^HSV^. Expectedly, the results revealed that the living T cells maintained their ability to secrete Granzyme B (Fig. S1b). Importantly, the infection with HSV-1^dko^-GFP had only minimal impact on the survival of CAR-T cells within the 3-day period following infection (Fig. 1h). Furthermore, we conducted an analysis of the CD8+/CD4+ ratio, cytokine secretion, T cell phenotype and expression levels of exhaustion markers to assess the effects of HSV-1^dko^-GFP infection. Intriguingly, after 24 h of HSV-1^dko^-GFP infection, there was a promotion of CD8-positive CAR-T cells (Fig. 1i), along with an increase in granzyme B secretion (Fig. 1i). However, the phenotype of T cells (CD44, CD62L) and the expression levels of PD1, TIM3, LAG3 did not exhibit any statistically significant differences (Fig. S2a, b, c and Fig. 1k). These findings suggest that a short-term infection with HSV-1^dko^-GFP does not adversely affect CAR-T cell functionality. Moreover, there appears to be a potential 3-day window for the delivery of HSV via CAR-T^HSV^ into tumor cells.

### Efficient delivery of HSV by *CAR*-T cells

**Fig. 2:**
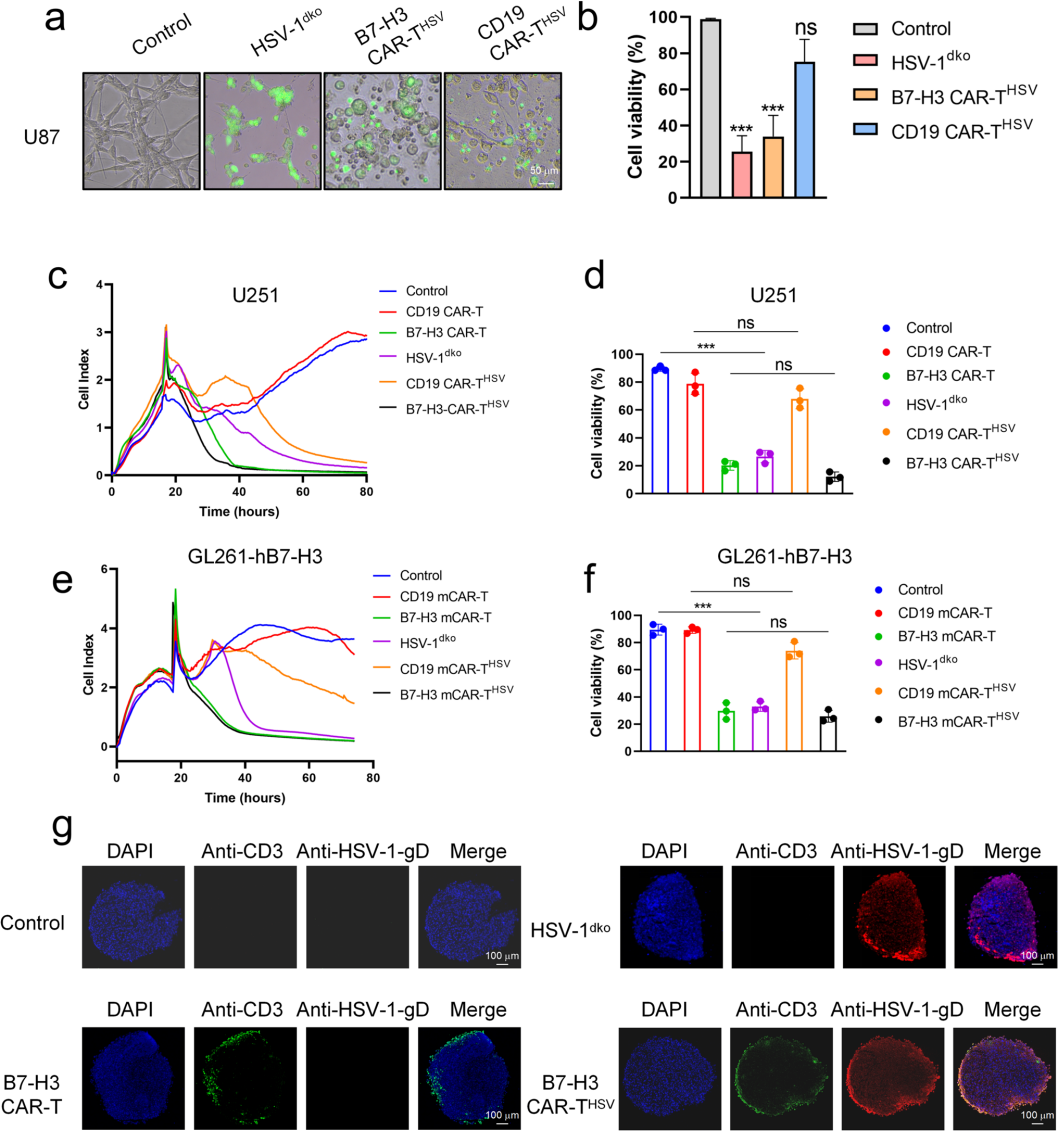
B7-H3 CAR-T^HSV^ cells display strong antitumor efficacy in vitro. **a** Representative images shown that B7-H3 CAR-T^HSV^ cells (5 × 10^3^) were cocultured with U87 tumor cells (1 × 10^4^) 24 h. **b** Statistical analysis of (**a**) showed that three random images from each group were selected to measure the viability cells, and the data are presented as mean ± s.d. ***P < 0.001 by unpaired two-tailed t-test. **c** and **e** the cytotoxicity of B7-H3 CAR-T^HSV^ cells or B7-H3 mCAR-T^HSV^ cells on U251 or Gl261-hB7-H3 cells in this coculture assay was monitored using the RTCA system. After an approximate time period of 18 to 20 h, CAR-T cells or CAR-T^HSV^ cells were introduced at a 4:1 effector-to-target ratio. **d** and **f** post 48-h coculture with U251 cells or GL261-hB7-H3 cells, B7-H3 CAR-T^HSV^ cells or B7-H3 mCAR-T^HSV^ cells were evaluated for their efficacy in killing tumor cells. n = 3/group. Values are presented as mean ± SD. ***P < 0.001, one-way ANOVA with Tukey test. **g** U251 spheroids were subjected to treatment with B7-H3 CAR-T^HSV^ cells, B7-H3 CAR-T cells, HSV-1^dko^, or a saline control for 24 h. Following this, paraffin sections of the U251 spheroids were prepared using paraformaldehyde fixation and subsequent paraffin embedding. The paraffin sections were processed for CD3 (green) and HSV-1-gD (red) double immunofluorescence staining. Pictures were captured by a confocal microscope (Zeiss 880), and the scale bars are 100 µm

Subsequently, we conducted experiments to assess the efficacy of B7-H3 CAR-T^HSV^ cells in eradicating tumor cells and effectively delivering HSV-1^dko^-GFP into the tumor cells, thereby demonstrating the crucial role of CAR-T cell tumor specificity in facilitating such a targeted delivery mechanism. U87 and U251, human glioblastoma cell lines, displayed B7-H3 high expression and CD19 negative (Fig. S3). As depicted in Fig. 2a, b, B7-H3 CAR-T^HSV^ cells, unlike CD19 CAR-T^HSV^ cells, efficiently delivered HSV-1^dko^ into U87 cells and hindered cell proliferation. Correspondingly, the Real-Time Cellular Analysis (RTCA) results indicated a similarity in the antitumor efficiency of B7-H3 CAR-T^HSV^ cells relative to B7-H3 CAR-T cells, but an enhancement relative to HSV-1^dko^ (Fig. 2c, d). Post 48-h coculture with U251 cells, CD19 CAR-T^HSV^ cells manifested moderate antitumor effects due to the released HSV-1^dko^ from CD19 CAR-T cells. Similar experimental findings were observed in the co-culture system utilizing mouse-derived CAR-T cells (B7-H3 mCAR-T cells) loaded with HSV-1^dko^ in conjunction with GL261-hB7-H3 cells (Fig. 2e, f). Additionally, we conceived a three-dimensional (3D) spheroid model of U251 cells to gauge the antitumor effectiveness of B7-H3 CAR-T^HSV^ cells. CD3 and HSV-1-gD double immunofluorescence staining of representative slices from B7-H3 CAR-T^HSV^-treated tumor spheroids demonstrated successful targeting of tumor spheroids by B7-H3 CAR-T^HSV^ cells, and the peripheral rim of tumor spheroid regions being infected by HSV-1^dko^ (Fig. 2g). These findings underscore the proficient delivery of CAR-T^HSV^ cells to target cancer cells in an antigen-dependent modality.

### The intravenous administration of HSV is prone to hepatic interception

Prior to the official in vivo experiment, we conducted an initial evaluation by intravenously administering different titers of HSV-Luci to observe the spatial dispersion of the virus within the living organism. In this study, we pre-implanted 1 × 10^5^ U87 cells into immunodeficient mice intracranially, seven days prior to the experimental procedures. Following the injection of HSV-Luci into mice 48 h, the virus distribution was confirmed by detecting bioluminescent signals. It was observed that with an increasing quantity of injected HSV-Luci viruses, the bioluminescent signal became more prominent specifically in hepatic tissues (Fig. S4a, b). This observation was further substantiated by the detection of bioluminescent signals in isolated organs, namely the heart, liver, spleen, lungs, and kidneys (Fig. S4c). Hence, based on these experimental findings, it can be deduced that the administration of HSV-Luci via tail vein injection primarily results in its enrichment within the liver.

### B7-H3 *CAR*-THSV targeted killing of the tumor cells in an immunodeficient mouse model

**Fig. 3:**
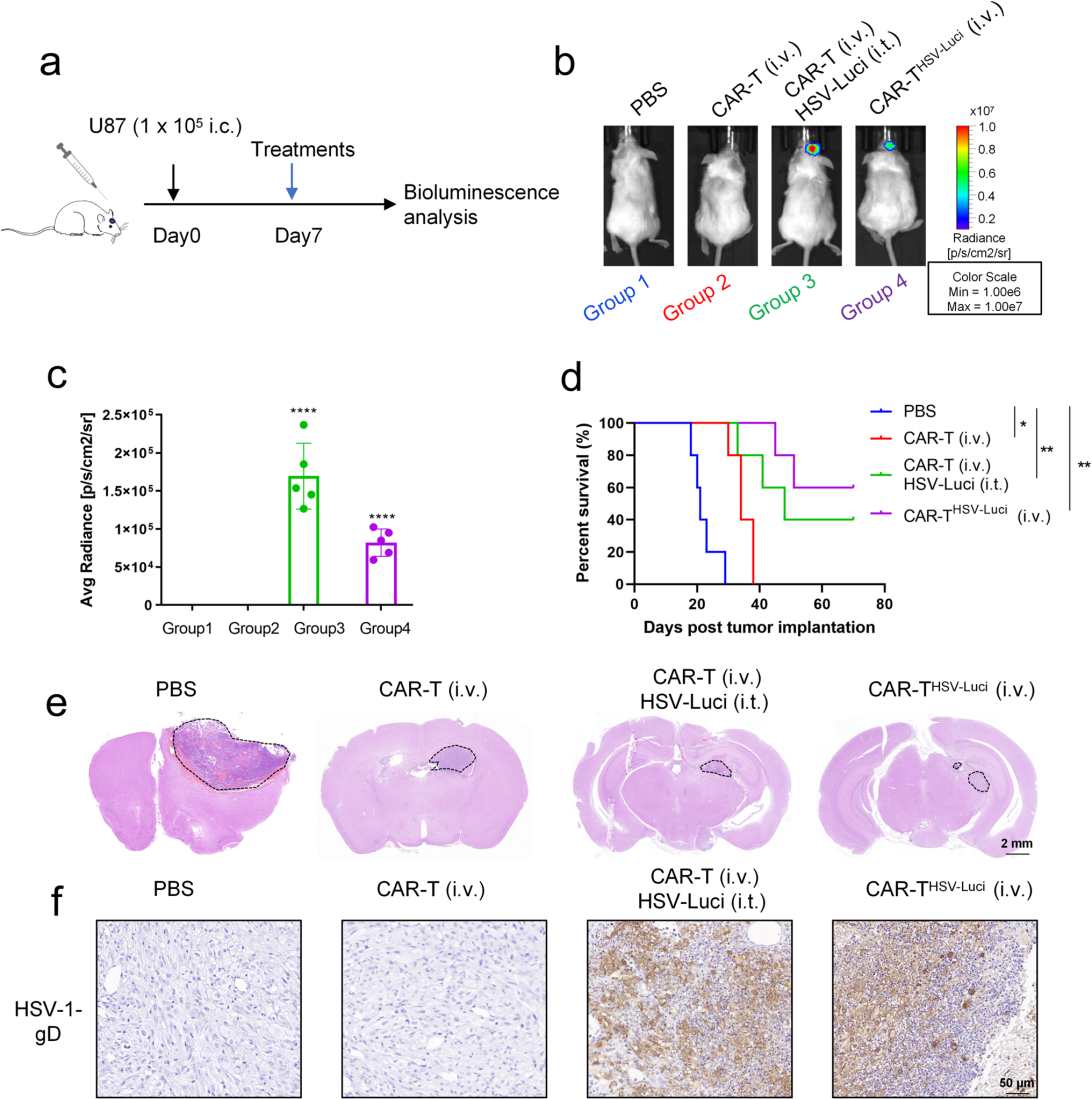
CAR-T^HSV^ cells successfully delivered HSV systemically in an immunodeficient mouse model. **a** Timeline of mouse models of U87 tumor cells with treatment schedules. On day 0, 1 × 10^5^ U87 GBM cells were intracranial administered into NCG mice. On day 7, they were differently administered with HSV, CAR-T cells or vehicle control. Group 1 received PBS (i.t.); Group 2 received B7-H3 CAR-T (i.v., 5 × 10^6^ cells); Group 3 received B7-H3 CAR-T (i.v., 5 × 10^6^ cells) in combination with HSV-Luci (i.t.); and Group 4 received B7-H3 CAR-T^HSV−Luci^ (i.v., 5 × 10^6^ cells). Bioluminescence was measured on day 10. Representative images (**b**) and summarized data are shown (**c**) (n = 5/group). Data are presented as mean values ± SD and were analyzed by Student’s t test. ****P < 0.0001. **d** Survival time of U87 tumor-bearing mice was analyzed using the Kaplan–Meier method with the log-rank test (n = 5 for each group). *P < 0.05; **P < 0.01. **e** H&E staining of the specimen isolated from experimental mice (scale bar, 2 mm). The demarcated regions symbolize the presence of tumors (marked in dashed line). **f** HSV infection 7 days post-treatment was confirmed by immunohistochemistry (scale bars, 50 µm)

Subsequently, we proceeded with evaluating the in vivo effectiveness of systemically delivering B7-H3 CAR-T^HSV^ cells by employing two mouse models. Initially, we employed a well-established orthotropic model of human GBM by intracranially injecting 1 × 10^5^ U87 cells into immunodeficient NCG mice (Fig. 3a). The mice were divided into four groups to receive either intravenous (i.v.) or intratumoral (i.t.) administration on day 7: Group 1 received PBS as a control; Group 2 received B7-H3 CAR-T (i.v.); Group 3 received B7-H3 CAR-T (i.v.) in combination with HSV-Luci (i.t.); and Group 4 received B7-H3 CAR-T^HSV−Luci^ (i.v.). As expected, the transfer of CAR-T^HSV−Luci^ cells successfully delivered HSV-Luci systemically in vivo following the intravenous injection of B7-H3 CAR-T^HSV−Luci^ cells into U87-bearing NCG mice (Fig. 3b, c). Both the i.t. HSV-Luci and i.v. B7-H3 CAR-T^HSV−Luci^ cell groups exhibited the ability to infect U87 tumors. Mice treated with saline control showed rapid tumor progression and succumbed to mortality within a span of 30 days, while the combination treatment in Group 3 and Group 4 exhibited a significant increase in their median survival time (Fig. 3d).

Simultaneously, during the progression of the study, the mice subjected to combination treatment exhibited a gradual and consistent increase in body weight compared to the other groups (Fig. S5a). After 18 days, the brains of the experimental mice were dissected for histological analysis. H&E staining of representative tissue slices from saline-treated mice revealed sizable, occupying tumors, whereas mice treated with B7-H3 CAR-T displayed a moderate degree of tumor regression. Remarkably, mice treated with B7-H3 CAR-T (i.v.) combined with HSV-Luci (i.t.) or single administration of B7-H3 CAR-T^HSV−Luci^ (i.v.) exhibited significantly tumor regression (Fig. 3e). Immunohistochemical staining with an anti-HSV-1-gD antibody demonstrated areas of HSV infection, with detectable HSV-1-gD protein in mice treated with HSV-Luci (i.t.) as well as those treated with B7-H3 CAR-T^HSV−Luci^ (i.v.) (Fig. 3f). We conducted a quantitative analysis to ascertain the comparative efficacy of HSV infection between groups 3 and 4. Regrettably, it was observed that the former exhibited a greater abundance of positive areas indicating HSV-gD presence. (Fig. S5b).

### *CAR*-T cells possess the capability to deliver the HSV to the tumor site in immunocompetent mouse model

**Fig. 4:**
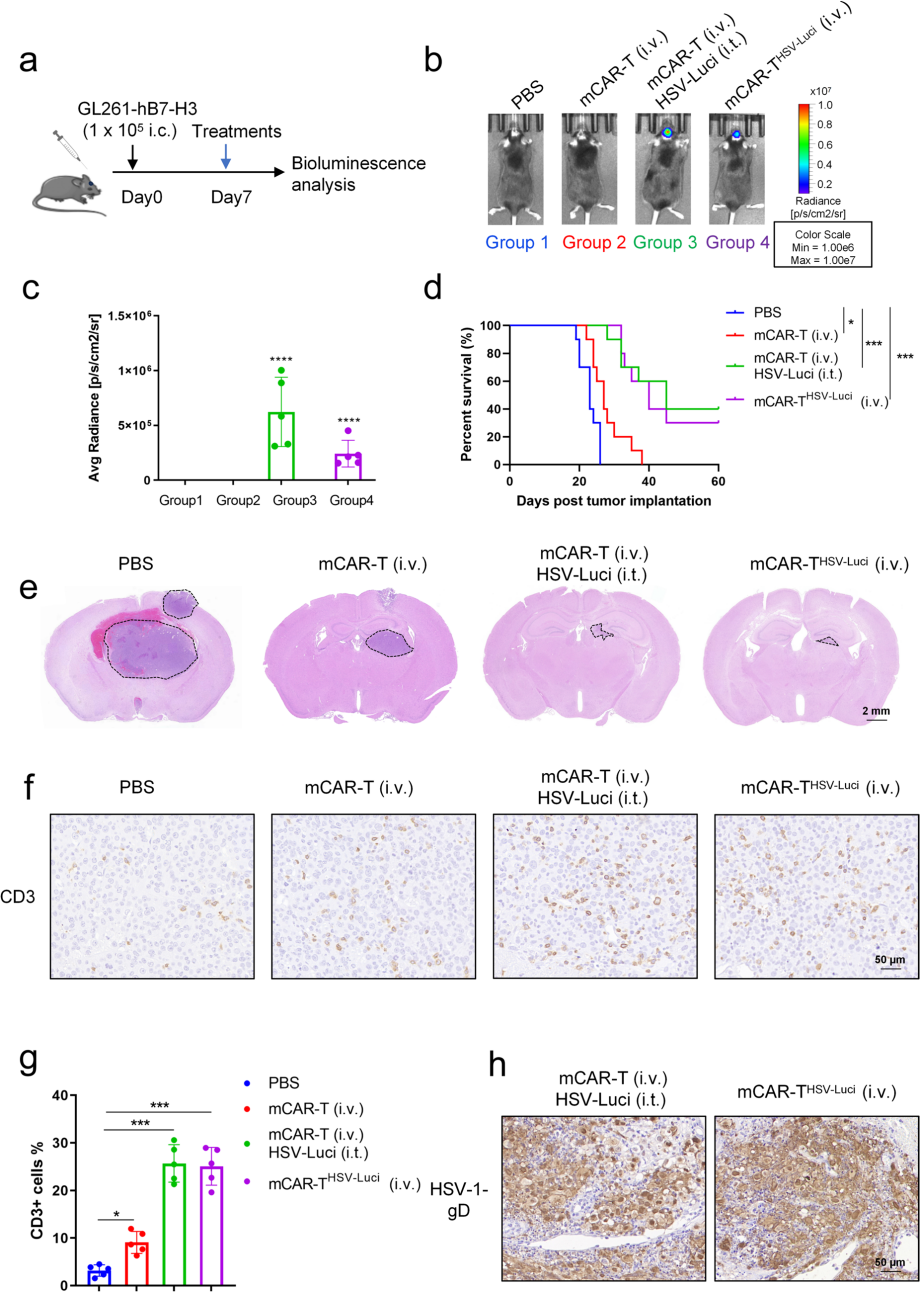
Murine CAR-T^HSV^ cells effectively delivered HSV to the tumor site of orthotopic GBM. **a** The orthotopic model of GBM in mice was established through intracranial injection of 1 × 10^5^ GL261-hB7-H3 cells into immunocompetent C57BL/6 mice. Seven days later, mice were injected with a vehicle control or HSV-Luci, or mCAR-T cells. **b** In groups 1, 2, and 3, PBS or HSV-Luci was i.t. injected into tumors. In groups 2, 3 and 4, B7-H3 mCAR-T or B7-H3 mCAR-T^HSV−Luci^ cells (5 × 10^6^ cells) were i.v. injected. Bioluminescence was measured on day 10. **b** Representative bioluminescence images are shown. **c** HSV-Luci infection within the tumors was monitored, and the average radiance (p/s/cm^2^/sr) was calculated (n = 5/group). Data are presented as mean values ± SD and were analyzed by Student’s t test. ****P < 0.0001. **d** Overall survival analysis of GL261-hB7-H3 tumor-bearing mice in each group (n = 10 per group). *P < 0.05; ***P < 0.001. Survival analysis was conducted by log-rank test. **e** H&E staining of sections of brains harvested from mice on day 18 after B7-H3 mCAR-T^HSV−Luci^ treatment. Representative images are shown. The delineated areas, marked with dashed lines, represent the presence of tumors. **f**, **h** Paraffin tumor sections were stained with CD3 and HSV-1-gD. Scale bar: 50 μm. **g** The CD3+ cells of per field in groups with different treatments were counted. Each group consisted of five mice, and one field was selected for assessment per mouse. The data are presented as mean values ± SD and were analyzed by Student’s t test. *P < 0.05; ***P < 0.001

In addition, to account for the immune system deficiency in NCG mice, an immunocompetent GBM mice model was employed to assess the efficacy of CAR-T cells in delivering HSV. B7-H3-targeted murine CAR-T (mCAR-T) cells were generated, encompassing a B7-H3-specific single-chain variable fragment (scFv), a transmembrane region derived from murine CD8, murine 4-1BB, murine CD3ζ intracellular regions, and a truncated form of murine CD34, following the previously reported protocol [33]. Subsequently, we conducted an antitumor study using the orthotopic model of mouse GBM by intracranially injecting 1 × 10^5^ GL261-hB7-H3 cells (mouse GBM cells expressing human B7-H3 gene) into immunocompetent C57BL/6 mice (Fig. 4a). Two days prior to treatment, lymphodepletion was induced by subjecting the mice to 5 Gy total body irradiation (TBI). Similarly, four different treatment groups were established, beginning 7 days after tumor inoculation: Group 1 received PBS as a control; Group 2 received B7-H3 mCAR-T (i.v.); Group 3 received B7-H3 mCAR-T (i.v.) in combination with HSV-Luci (i.t.); and Group 4 received B7-H3 mCAR-T^HSV−Luci^ (i.v.). As anticipated, B7-H3 mCAR-T^HSV−Luci^ demonstrated the capability to deliver HSV-Luci into tumor cells (Fig. 4b). The bioluminescent signal originating from HSV-Luci was detected, indicating the presence of HSV-Luci infection within the tumors. Both the i.t. HSV-Luci and i.v. B7-H3 mCAR-T^HSV−Luci^ groups exhibited bioluminescent signals, with the former demonstrating stronger signals (Fig. 4c).

Nonetheless, the combined treatment of B7-H3 mCAR-T (i.v.) along with HSV-Luci (i.t.) or B7-H3 mCAR-T^HSV−Luci^ (i.v.) demonstrated a notable antitumor efficacy in the immunocompetent mouse model, comparable to the treatments employed in the immunodeficient mouse model. Both treatment strategies exhibited a significant prolongation of survival compared to the saline control (Fig. 4d), as well as a gradual and consistent increase in body weight in contrast to the other groups (Fig. S6a).

The isolated organs including the heart, liver, spleen, lungs, kidneys and brains from the experimental mice were subjected to histological examination. H&E staining was conducted to differentiate GBM tissues from normal brain tissues (Fig. 4e). Notably, based on the HE staining results of this particular brain tissue section, it could be inferred that mice treated with B7-H3 mCAR-T (i.v.) in combination with HSV-Luci (i.t.) or B7-H3 mCAR-T^HSV−Luci^ (i.v.) demonstrated a significant regression of tumors. From the histological examination of the heart, liver, spleen, lung, and kidney tissues stained with H&E, we did not observe any signs of tissue damage or viral accumulation in mice that received intravenous injections of CAR-T^HSV^ cells (Fig. S7). Immunohistochemical staining with an anti-HSV-gD antibody or an anti-CD3 antibody demonstrated the presence of HSV-infected regions and the distribution of mCAR-T or T cells, respectively (Fig. 4f–h). It was observed that the co-administration of B7-H3 mCAR-T (i.v.) with HSV-Luci (i.t.) exhibited a greater abundance of positive areas indicating HSV-gD presence (Fig. S6b). Analysis of CD3 staining indicated that while T cells infiltrated minimally into the tumor upon treatment with B7-H3 mCAR-T (i.v.) alone, the combination treatment of B7-H3 mCAR-T (i.v.) with HSV-Luci (i.t.) or B7-H3 mCAR-T^HSV−Luci^ (i.v.) significantly increased intratumoral T cell infiltration (Fig. 4f, g). These data suggest that both B7-H3 mCAR-T (i.v.) in conjunction with HSV-Luci (i.t.) and B7-H3 mCAR-T^HSV−Luci^ (i.v.) treatments leads to significant enhancements in therapeutic outcomes. Moreover, it is evident that HSV can be effectively systemically delivered by B7-H3 CAR-T cells.

### *CAR*-THSV^−Luci^ cells exhibit notable advantages in the therapeutic management of distal tumors

**Fig. 5:**
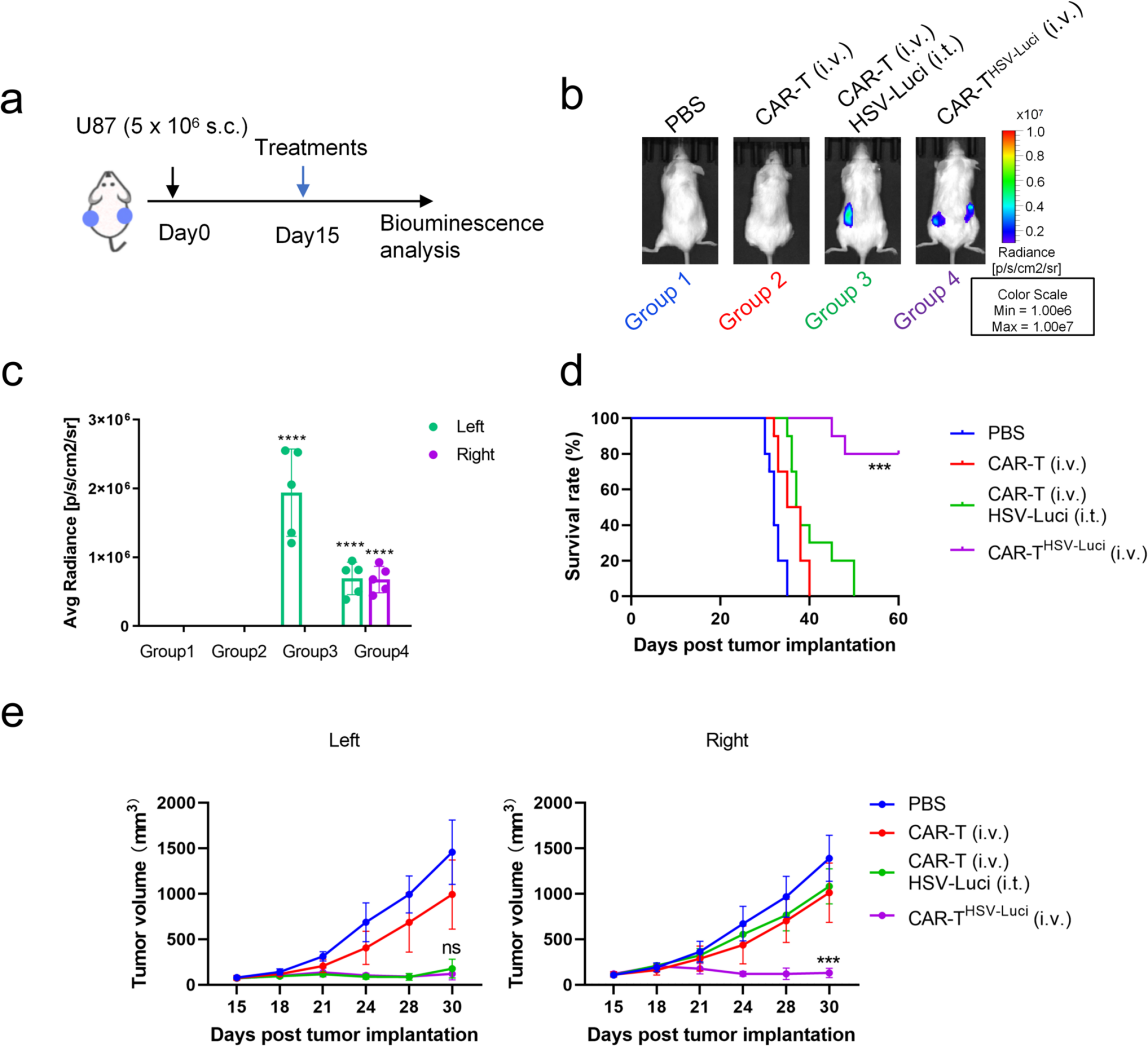
The CAR-T^HSV^ cells confers distinct advantages in systematically delivering HSV to distant tumors. **a** NCG mice were subjected to s.c. inoculation of 5 × 10^6^ U87 cells bilaterally on their flank regions. On day 15, mice were treated when tumors reached approximately 80 mm^3^. **b** In groups 1, 2, and 3, PBS or HSV-Luci was i.t. injected only into tumors on the left flank, and tumors on the right flank did not receive an i.t. injection. In groups 2, 3 and 4, B7-H3 CAR-T or B7-H3 CAR-T^HSV−Luci^ cells (5 × 10^6^ cells) were i.v. injected. Bioluminescence was measured on day 18, and summarized data are shown (**c**). Data are presented as mean values ± SD and were analyzed by Student’s t test. ****P < 0.0001, group 3 (left) compared with groups 1 and 2 (left); ****P < 0.0001, group 4 (left) compared with groups 1 and 2 (left); ****P < 0.0001, group 4 (right) compared with groups 1 and 2 (right). **d** An analysis of overall survival was conducted on a group of mice bearing s.c. U87 tumors, with a total of 10 mice in each group. ***P < 0.001. Survival analysis was conducted by log-rank test. **e** Left and right tumor growth in the control group 1, B7-H3 CAR-T (i.v.) group 2, B7-H3 CAR-T (i.v.) in combination with HSV-Luci (i.t.) group 3 and B7-H3 CAR-T^HSV−Luci^ (i.v.) group 4 (n = 5 mice per group). Data are mean ± SD. ns, group 4 (left) compared with groups 3 (left); ***P < 0.001, group 4 (right) compared with groups 3

While intratumoral administration of HSV represents an elective amalgamation strategy within CAR-T therapy, possessing the capacity to reprogram the immunosuppressive tumor microenvironment (TME) and augment infiltration of CAR-T cells, it is plausible that tumor cells outside the ambit of direct viral injection, including disseminated metastatic tumors, evince limited susceptibility to HSV injection, thereby impeding the potential synergy between HSV and CAR-T cells in the collective endeavor to effectuate tumor eradication. Employing CAR-T cells as a vehicle for delivering HSV to tumors may serve as a potential solution to circumvent this challenge. Here, NCG mice were utilized to examine this hypothesis, with the mice receiving subcutaneous (s.c.) injections of 5 × 10^6^ U87 cells on both sides of their flanks (Fig. 5a). As depicted in Fig. 5b, mice that received i.t. administration of HSV-Luci solely into the tumors inoculated on one side did not observe viral spread to tumors located on the non-injected side. However, remarkably, i.v. transfer of B7-H3 CAR-T^HSV−Luci^ cells effectively facilitated the delivery of HSV-Luci to U87 tumors present on both sides of the mice. This was evidenced by a significant increase in bilateral bioluminescence signals, highlighting the successful delivery of HSV-Luci to the tumors (Fig. 5b, c). Based on the results obtained from group 3, the i.t. injection of HSV-Luci into tumors located on the left flank of mice, when combined with i.v. administration of B7-H3 CAR-T cells, demonstrated a significant inhibition of tumor growth specifically in the left flank, surpassing the impact observed in the i.v. B7-H3 CAR-T cell group alone. However, it is worth noting that the right flank tumors did not exhibit any discernible improvement in response without the injection of HSV-Luci (Fig. 5e). In contrast, the i.v. administration of B7-H3 CAR-T^HSV−Luci^ cells exhibited a superior response in inhibiting the growth of tumors on both flanks, demonstrating robust antitumor efficacy and leading to long-term survival (Fig. 5d, e). Our findings emphasize the significance of combining therapy involving the i.t. injection of HSV and i.v. administration of B7-H3 CAR-T cells, as it substantially improves the responsiveness of left-flank tumors that were injected with HSV. However, this combination therapy does not yield improved results on right-flank tumors without HSV injection. Nevertheless, the introduction of i.v. B7-H3 CAR-T^HSV−Luci^ cells successfully overcomes these obstacles and achieves positive outcomes on both flanks.

## Discussion and conclusion

In this study, a novel strategy was developed for the systemic delivery of oncolytic HSV into solid tumors through the transfer of B7-H3 CAR-T^HSV^ cells. The capability of B7-H3 CAR-T cells to deliver HSV to solid tumors was demonstrated using both immunodeficient and immunocompetent orthotopic GBM mouse models. Additionally, a bilateral subcutaneous tumor inoculation model was employed to evaluate the responsiveness of the left-flank tumors, which were injected with HSV, and the non-injected right-flank tumors when combined with intravenous administration of B7-H3 CAR-T cells. The combination of i.t. HSV injection and i.v. administration of B7-H3 CAR-T cells significantly enhanced the responsiveness of the left-flank tumors that received HSV injection, while no observable improvement was seen in the right-flank tumors that did not receive HSV injection. Notably, B7-H3 CAR-T cells carrying HSV demonstrated significant inhibition of bilateral tumor growth in mice according to this model. To the best of our knowledge, this is the first study to assess the CAR-T cell delivery of an engineered oncolytic HSV. Our study uncovered that this delivery modality can be possible to translate into clinical practice for solid tumor types characterized by disseminated metastatic features and effective targeting by B7-H3 CAR T cells.

Oncolytic viruses (OVs) are emerging as promising therapeutic agents for cancer treatment due to their unique ability to selectively replicate within and lyse cancer cells, while sparing normal cells [22, 23]. Among the currently available OV therapies, the HSV-1-based T-Vec is the only one that has received FDA approval for the treatment of advanced melanoma, and it is administered through local intratumoral delivery [34]. Extensive clinical and preclinical investigations have demonstrated the safety and efficacy of localized intratumoral delivery OVs, resulting in positive antitumor responses in specific cases.

Recently, the systemic delivery of OVs to tumors has garnered considerable attention. Several strategies have been devised to augment the systemic delivery of OVs. One strategy entails the genetic manipulation of OVs to selectively target tumor cells through the incorporation of tumor-specific ligands or antibodies on the viral surface [35–37]. This modification enables the preferential binding and internalization of OVs into cancer cells, thereby enhancing their therapeutic effectiveness. Another strategy involves the integration of OVs with carrier systems such as nanoparticles, liposomes, or exosomes, which confer protection, stability, and improved tumor accumulation of OVs during systemic administration [38–43]. However, the systemic delivery of OVs remains a significant challenge due to various immune barriers and tumor microenvironment factors. The innate and adaptive immune systems play a crucial role in effectively defending the host by primarily eliminating circulating naked virions before they can reach a tumor [14, 23]. It is widely acknowledged that enhancing the delivery system for OVs is crucial for boosting their therapeutic efficacy, particularly in combatting metastatic or diffusely infiltrating tumors. Using autologous host mammalian cells or host-derived engineered cells can prevent recognition by the host immunity, which makes it possible to conceal an oncolytic virus within these cells [44]. This approach presents a potential solution for overcoming the elimination of systemically delivered OVs. Consequently, the delivery of oncolytic viruses via carrier cells to tumor cells becomes a viable strategy. Ideally, the carrier cells should possess the ability to target or home to the specific tumor site. Some studies have shown that mesenchymal stem cells (MSCs) can successfully transport oncolytic viruses, such as attenuated measles virus, adenovirus, and herpes simplex virus, to target cells [45–47]. Nevertheless, there is a scarcity of reports regarding the effectiveness of CAR-T cells as carriers for delivering oncolytic viruses specifically aimed at targeting and eliminating tumors [24, 25]. One recent study has been conducted thus far to examine the potential exploitation of CAR-T and TCR-T cells as carrier cells for delivering myxoma virus [48].

An efficient cellular delivery system should possess the capability to sustain its own viability within a specific timeframe, while also generating a significant number of infectious offspring upon reaching the tumor site [44, 49]. We found that the activity of CAR-T cells infected with HSV had not been obvious effect within 3 days (Fig. 1h). Therefore, the survival time of a virus-loaded CAR-T cell is limited. In our study, CAR-T^HSV^ cells accumulate around tumor spheroid and released sufficient amounts of infectious progeny to induce oncolysis on tumor spheroid after cocultured 24 h (Fig. 2g). Similarly, intravenous administrations of virus-loaded CAR-T cells reach the tumors after 24 h, and HSV initiate the process of replication that leads to progeny virion production and release in tumors. Moreover, according to our in vivo observation, CAR-T^HSV^ cells produced high intratumoral levels of progeny at the tumor site that were similar to naked HSV injection (Figs. 3f, 4h). Hence, three days was enough for CAR T cells to reach the tumor site and release the virus. Intriguingly, infection with HSV may have somehow improved the effector functions of CAR-T^HSV^ cells because we observed increased Granzyme B production after loaded with HSV 24 h (Fig. 1j). We hypothesized that CAR-T^HSV^ plus non-infected CAR-T cells might achieve better tumor-killing effects, because CAR-T^HSV^ loads HSV by 13.69%, and granzyme B secreted by CAR-T cells loaded with HSV is able to promote tumor killing by non-infected CAR-T cells. Undoubtedly, further research and careful consideration are necessary to conduct thorough testing and verification.

In conclusion, our study has demonstrated the significant improvement in efficacy and biodistribution profile of anti-glioma oncolytic virotherapy using B7-H3 CAR-T cell carrier HSV in an animal model. This carrier system exhibits targeted tumor cell delivery while preserving the antitumor functionality of CAR-T cells over a specific duration. Furthermore, we conducted a comparative analysis involving both immunodeficient and immunocompetent orthotopic GBM mouse models to evaluate the effectiveness and biodistribution profiles of CAR-T cell-based oncolytic virotherapy. These findings provide valuable insights for researchers interested in leveraging the CAR-T cell carrier approach for precise oncolytic virus delivery to metastatic tumor loads in cancer patients, thereby facilitating the translation of CAR-T cell carrier-based oncolytic virotherapy to clinical settings.

### Supplementary Information

Below is the link to the electronic supplementary material.

Supplementary file1 (DOCX 2005 kb)

## Data Availability

Additional information and inquiries pertaining to resources and reagents ought to be directed towards the corresponding author, who will duly address and fulfill them.
