# Supplementary material for: Systemic delivery of oncolytic herpes virus using CAR-T cells enhances targeting of antitumor immuno-virotherapy
Source: Cancer Immunol Immunother. 2024 Jul 2;73(9):173. doi: 10.1007/s00262-024-03757-8 (PMC11219689; doi:10.1007/s00262-024-03757-8)
Supplement: Supplementary file 1 — Supplementary file1 (DOCX 2005 kb) [file 262_2024_3757_MOESM1_ESM.docx]

Supplementary information for

Systemic delivery of oncolytic herpes virus using CAR-T cells enhances targeting of antitumor immuno-virotherapy

Zongliang Zhang^1#^, Nian Yang^1#^, Long Xu^1#^, Huaqing Lu^1^, Yongdong Chen^1^, Zeng Wang^1^, Qizhong Lu^1^, Kunhong Zhong^1^, Zhixiong Zhu^1^, Guoqing Wang^2^, Hexian Li^1^, Meijun Zheng^3^, Liangxue Zhou^*4,5,6^, Aiping Tong^*1,7^

^1^State Key Laboratory of Biotherapy and Cancer Center, Research Unit of Gene and Immunotherapy, Chinese Academy of Medical Sciences, Collaborative Innovation Center of Biotherapy, West China Hospital, Sichuan University, Chengdu Sichuan Province, 610041 China.

^2^Department of Ophthalmology, West China Hospital, Sichuan University, West China Medical School, Chengdu, Sichuan, 610041, China

^3^Department of Otolaryngology, Head and Neck Surgery, West China Hospital, West China Medical School, Sichuan University, Chengdu, Sichuan, 610041, China

^4^Department of Neurosurgery, West China Hospital, West China Medical School, Sichuan University, Chengdu, Sichuan, 610041, China

^5^Department of Neurosurgery, Fifth People's Hospital of Ningxia Hui Autonomous Region, Shizuishan, Ningxia, 753000, China

^6^Department of Neurosurgery, Mianyang Central Hospital, Mianyang, Sichuan, 621000, China.

^7^Frontiers Medical Center, Tianfu Jincheng Laboratory, Chengdu 610212, China.

^#^These authors contributed equally to the manuscript.

^*^Corresponding Author:

Dr Aiping Tong; aipingtong@scu.edu.cn & Dr Liangxue Zhou; zhlxlll@163.com

1. Materials and Methods
2. Supporting figures and figure legends

**Materials and Methods**

**Animals**

Female mice, aged six to eight weeks, of the C57BL/6 and NCG (NOD/ShiLtJGpt-Prkdc^em26Cd52^II2rg^em26Cd22^/Gpt) strains, were obtained from Jiangsu GemPharmatech. The mice were housed in a specific pathogen-free environment, and all experiments were conducted with the approval of the Institutional Animal Care and Use Committee of Sichuan University.

**Cell lines and viruses**

The U87, U251 human glioblastoma (GBM) cell lines, and A375 human melanoma cell line were acquired from ATCC. The GL261-hB7-H3 murine GBM cell line, which expresses the human B7-H3 gene, was generated previously in our laboratory by transfecting the murine GL261 cell lines. Cells were cultured in DMEM supplemented with 10% FBS, penicillin (100 U/ml), and streptomycin (100 μg/ml). The U87 cells were maintained as tumor spheres in low-attachment cell culture flasks, using the basic neurobasal medium, at a density of 1,000 cells/ml. The HSV-1^dko^, obtained by deleting a 0.3 kb segment of the ICP47 gene, 0.8 kb segment of the ICP34.5 gene, and a 1 kb segment of the ICP6 gene in the HSV-1 (CCTCC NO: V202271) genome, was utilized. GFP or Luciferase (Luci) genes, under the control of the CMV promoter, were incorporated into both copies of the ICP34.5 regions of HSV-1^dko^. The quantification of HSV titers was performed using a foci formation assay on vero cells. In some experiments, treated CAR-T^HSV^ cells were lysed by subjecting them to three cycles of freezing and thawing, and the resulting cell lysates, containing the virus, were collected.

**CAR-T cell preparation**

As previously mentioned, human peripheral blood mononuclear cells (PBMCs) were isolated from a healthy donor. Subsequently, human T cells were activated by CD3/CD28 Dynabeads and stimulated with 100 U/mL hIL-2 for 48 hours. These activated T cells were then transduced with lentivirus encoding B7-H3-hBBZ-CAR or CD19-hBBZ-CAR, targeting human B7-H3 or CD19 respectively. The transduction process was carried out in plates pre-coated with 10 μg/mL RetroNectin (TaKaRa) through centrifugation at 1,800 rpm for 2 hours, maintaining a temperature of 32℃. Continuous culture of the transduced T cells was performed using X-vivo medium (Lonza) supplemented with IL-2 and IL15 (PeproTech). To track the expression of CAR, mCherry was inserted as a reporter, and its levels were measured using flow cytometry. The transduction rates of CAR vectors were approximately 50%.

Spleens from C57BL/6 mice were used to isolate murine T cells, which were subsequently activated using CD3/CD28 Dynabeads and stimulated with 100 U/mL hIL-2 for 48 hours. In a similar manner to human T cells, the activated murine T cells were transduced with retrovirus encoding B7-H3-mBBZ-CAR or CD19-mBBZ-CAR. The transduction was performed in plates pre-coated with 10 μg/mL RetroNectin (TaKaRa) through centrifugation at 1,800 rpm for 2 hours, maintaining a temperature of 32℃.

**Coculture assays**

The functionality of CAR-T^HSV^ cells was evaluated through coculturing them with various target cells such as U251, U87, A375, or GL261-hB7-H3 tumor cells. A total of 2 × 10^4^ CAR-T^HSV^ cells were mixed with an equal number of target cells in 96-well plates. After coculturing for 24 hours, 20 µl of MTT solution (5 mg/mL) was added and incubated for 4 hours. Subsequently, the medium was replaced with 150 µl of DMSO (Sigma-Aldrich), and the absorbance was measured at 570 nm using a microplate reader. Additionally, the coculture assays were simultaneously monitored using the xCELLigence® real-time cell analyzer (ACEA Biosciences). In this analysis, 4,000 tumor cells were initially seeded in each well for approximately 20 hours. Following this, CAR-T cells or CAR-T^HSV^ cells were added at a 4:1 effector-to-target ratio, and the cell index, representing cellular growth and viability, was measured every 30 minutes over a period of 80 hours. In the three-dimensional (3D) spheroid coculture model, U87 tumor spheroids were utilized. These spheroids were cocultured with vehicle control (PBS), HSV-1^dko^, B7-H3 CAR-T cells, or B7-H3 CAR-T^HSV^ cells for a duration of 24 hours.

**Flow cytometry and Immunofluorescence**

The infection efficiency of HSV-1^dko^-GFP on CAR-T cells was analyzed using a NovoCyte Flow Cytometer. Immunofluorescence analyses were conducted utilizing HSV-1^dko^-GFP-infected CAR-T cells after 48 hours. The cells were subjected to staining with antibodies specific for HSV-1-gD (Santa Cruz, clone DL6) in order to discern the localization of the virus. In a concise summary, the cells were fixed with 4% paraformaldehyde for 15 minutes at ambient temperature, then obstructed using a blocking buffer consisting of 5% normal serum and 0.1% Triton X-100 for 30 minutes. Subsequently, HSV-1-gD mouse monoclonal antibody was applied at a dilution of 1:200 and incubated with the cells for 2 hours at 37 ℃. This was followed by further incubation of the cells with goat anti-mouse IgG Alexa Fluor Cy3 Conjugate (Jackson ImmunoResearch). Visual documentation of the samples was achieved using a confocal microscope (Olympus IXplore SpinSR). Within the three-dimensional (3D) spheroid coculture model, spheroids were fixed in formalin, embedded in paraffin, and subsequently sectioned at a thickness of 4 μm. Immunofluorescence staining employing anti-HSV-1-gD and anti-CD3 antibodies was carried out, while the cell nuclei were visualized through staining with DAPI (Solarbio). Image acquisition was performed using a fluorescence microscope (Zeiss 880).

**Immunohistochemistry**

Tissue samples obtained from mice afflicted with GBM were immersed in formalin for preservation, subsequently fixed in paraffin, and sectioned at a thickness of 4 μm. H&E staining, along with immunohistochemical staining utilizing anti-HSV-1-gD and anti-CD3 antibodies, were executed. The stained slides were then mounted and examined using a fluorescence microscope (DP80, Olympus)

**Supporting figures and figure legends**

**
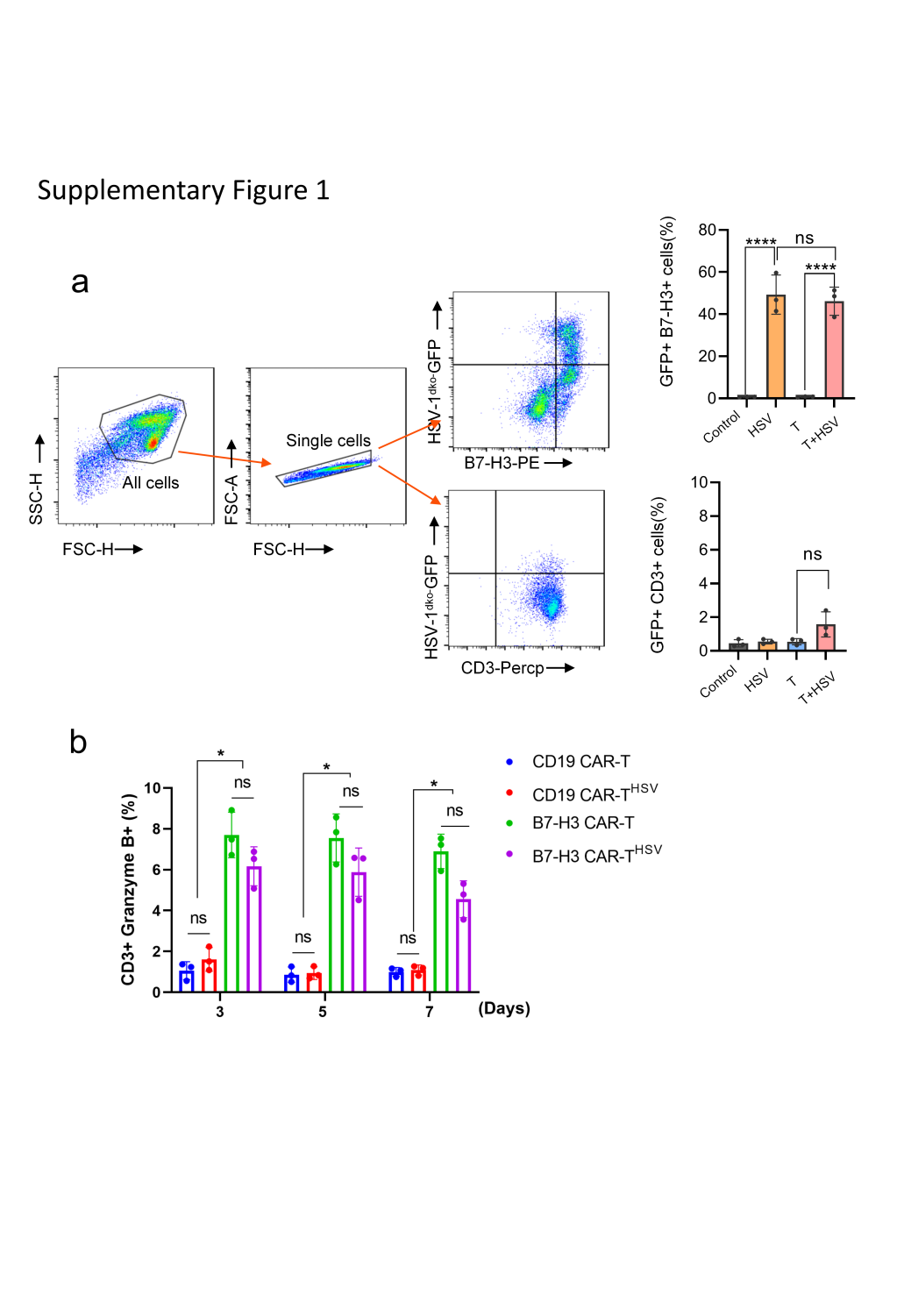
**

**Figure S1** (a) The efficiency of HSV virus infection in a co-culture system of U87 cells and T cells was assessed. Six-well plates were seeded with 1×106 U87 cells. After 12 hours, T cells were added at an effector-to-target ratio (E:T) of 1:1, along with the HSV-gfp virus (MOI=1). Following 48 hours of culture, flow cytometry was performed to assess the proportion of tumor cells expressing the surface marker B7-H3, T cells expressing CD3, and the percentage of cells positive for HSV-GFP infection. Representative images and percentages of GFP+ B7-H3+ U87 cells or GFP+ CD3+ T cells are shown (n = 3/group). Data are mean ± SD. ****P < 0.0001, ns, not significant; one-way ANOVA with Tukey test. (b) CAR-T cells were incubated with viruses for durations of 3, 5, and 7 days, after which they were co-cultured with tumor cells at an effector-to-target ratio of 4:1. The secretion levels of Granzyme B by viable T cells were subsequently quantified to evaluate the cytotoxic capacity of CAR-T^HSV^ (n = 3/group). Data are mean ± SD. *P < 0.05, ns, not significant; two-way ANOVA with Tukey test.

**
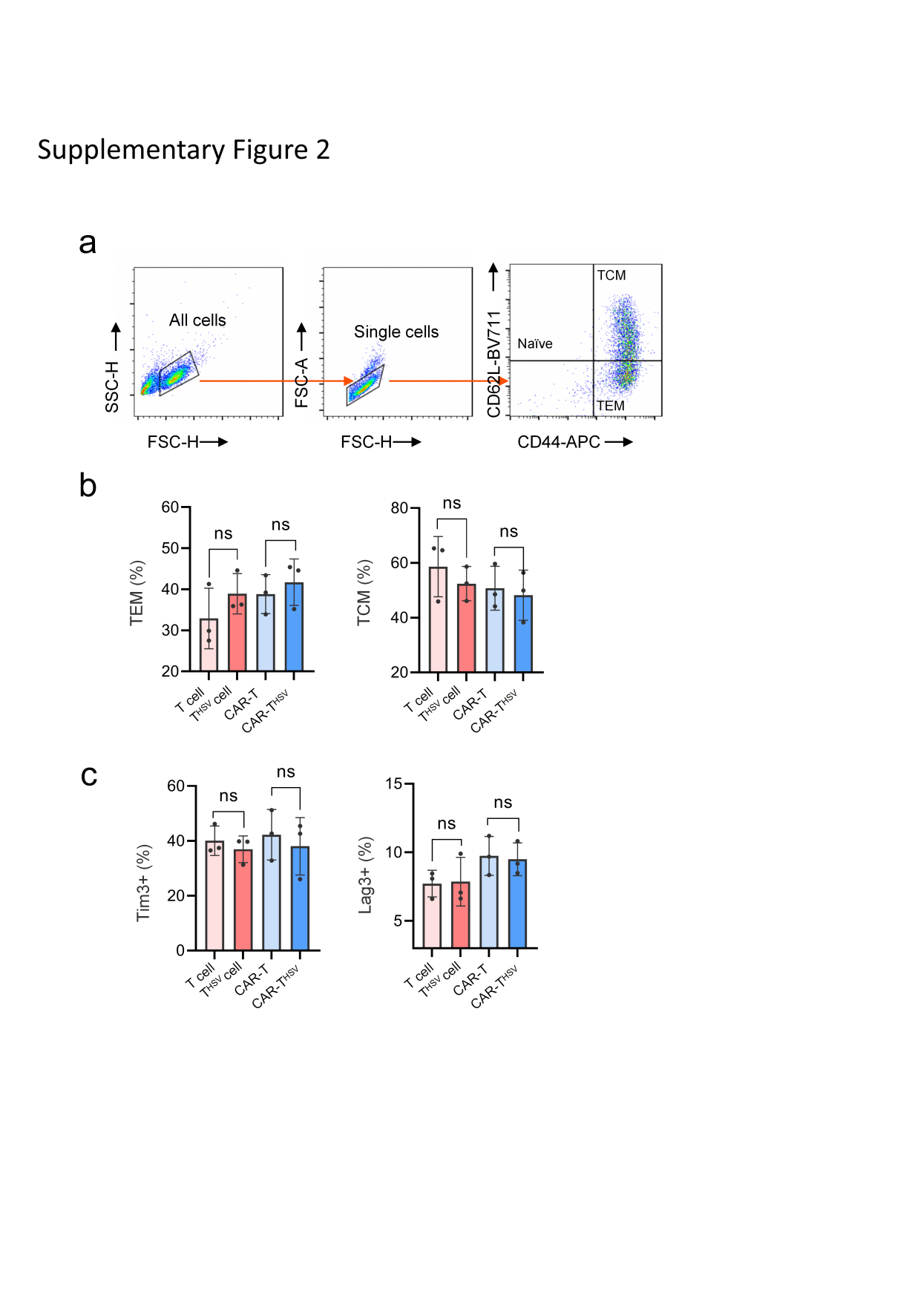
**

**Figure S2 Characterization of the CAR-T^HSV^ in vitro.**

After infecting HSV-1^dko^-GFP 24 hours, T cells or B7-H3 CAR-T cells were conducted to assess its properties and features. (a) A schematic illustration depicting the flow cytometry gating strategy employed in this experiment is presented. (b) The T cell phenotype markers, CD44-APC and CD62L-BV711, were assessed. (c) Furthermore, the exhaustion markers, Tim3-BV421 and Lag3-PE, were examined. n = 3/group. Values are presented as mean ± SD. ns, not significant; one-way ANOVA with Tukey test.

**
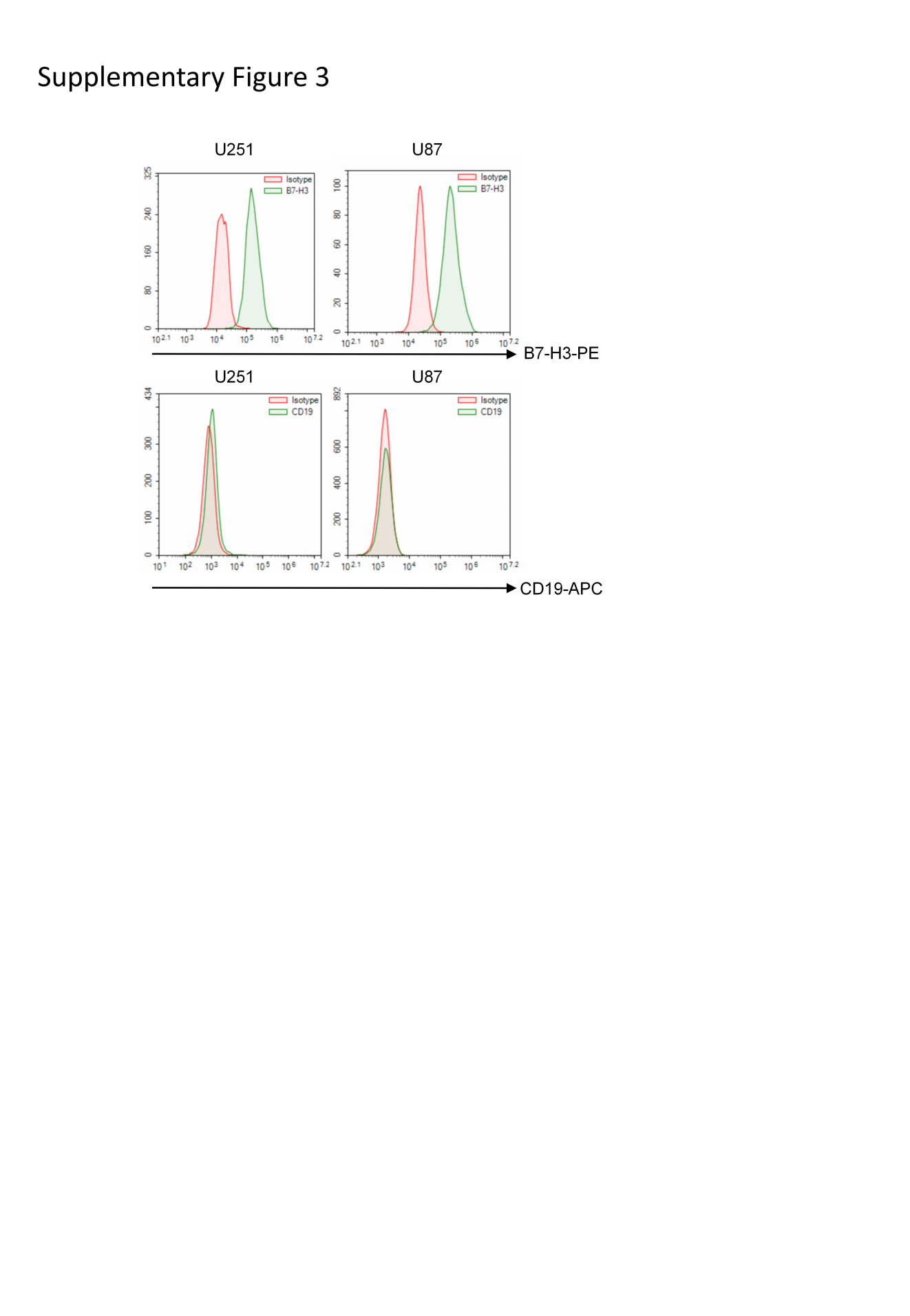
**

**Figure S3** Expression of B7-H3 and CD19 in U87 and U251 cell lines were evaluated by Flow cytometry. Cells were incubated with B7-H3-PE or CD19-APC (red) or its corresponding isotype control (green).

*
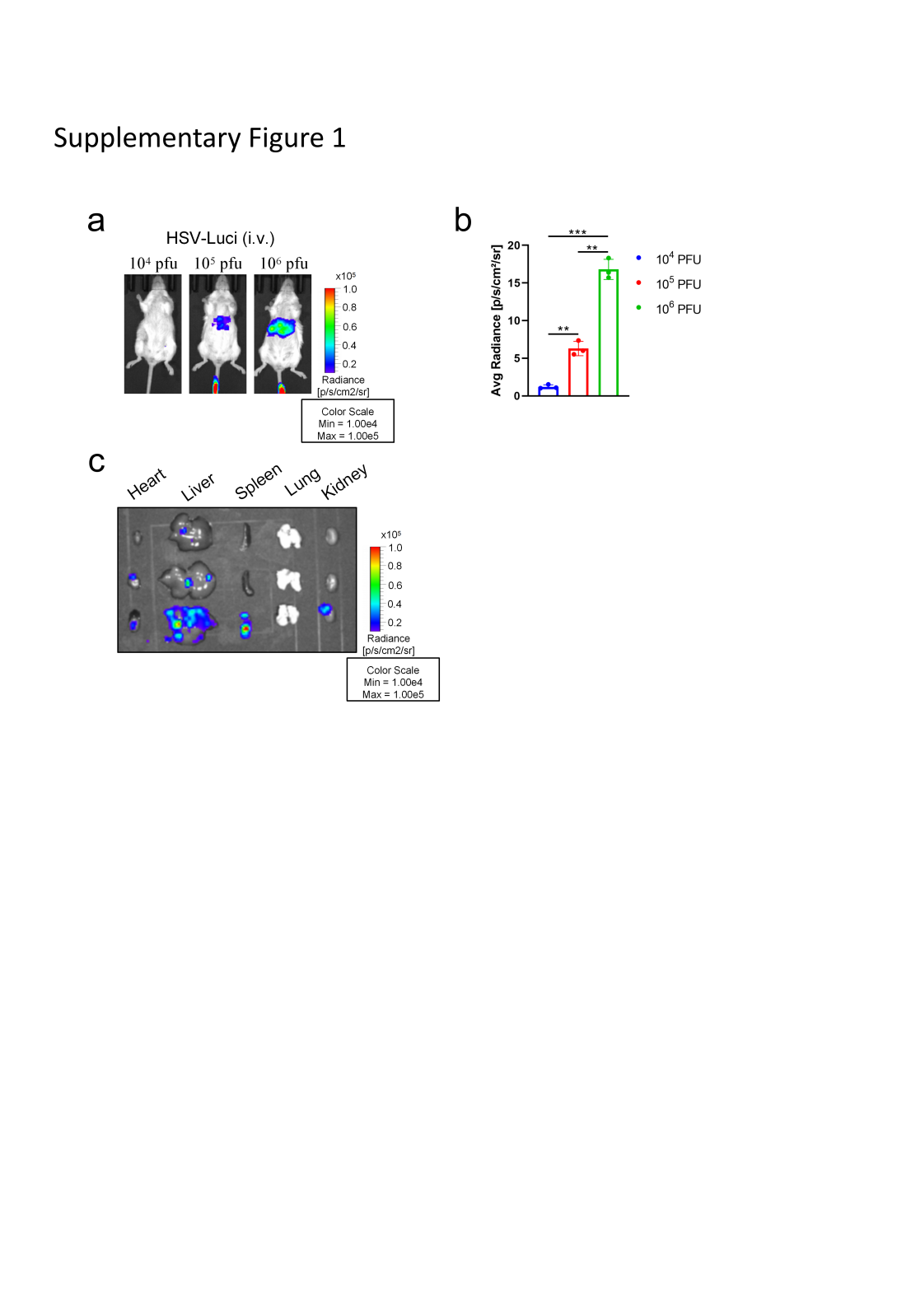
*

**Figure S4 The biodistribution of intravenously administered HSV at various titers in mice.**

(a) The bioluminescence images were collected at 48 h after intravenous injection of 10^4^, 10^5^, 10^6^ plaque forming unit (pfu) HSV. (b) The average tumor radiance (p/s/cm^2^/sr) was calculated (n = 3/group). Data are mean ± SD. **P < 0.01, ***p < 0.001. (c) Simultaneously, ex vivo bioluminescent images of heart, liver, spleen, lung, and kidney tissues were collected.

**
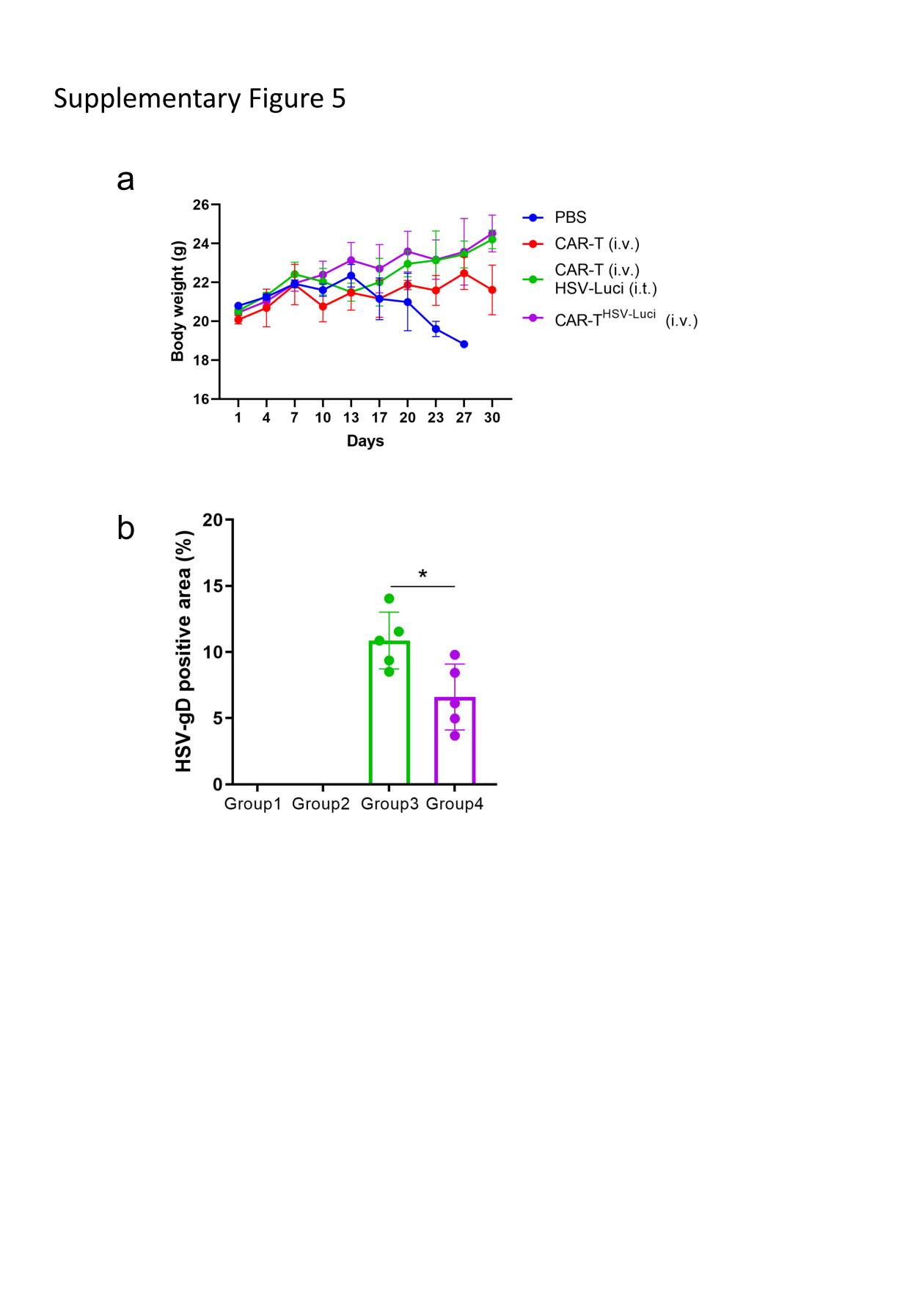
**

**Figure S5** (a) The body weight of each mouse was assessed at three-day intervals in the U87 tumor-bearing murine model. n = 5 mice per group. (b) The efficacy of HSV infection between groups 3 and 4 was compared (n = 5). Data are presented as mean values ± SD and were analyzed by Student’s t test. *P < 0.05. Group 1 received PBS as a control; Group 2 received B7-H3 CAR-T (i.v.); Group 3 received B7-H3 CAR-T (i.v.) in combination with HSV-Luci (i.t.); and Group 4 received B7-H3 CAR-T^HSV-Luci^ (i.v.).

*
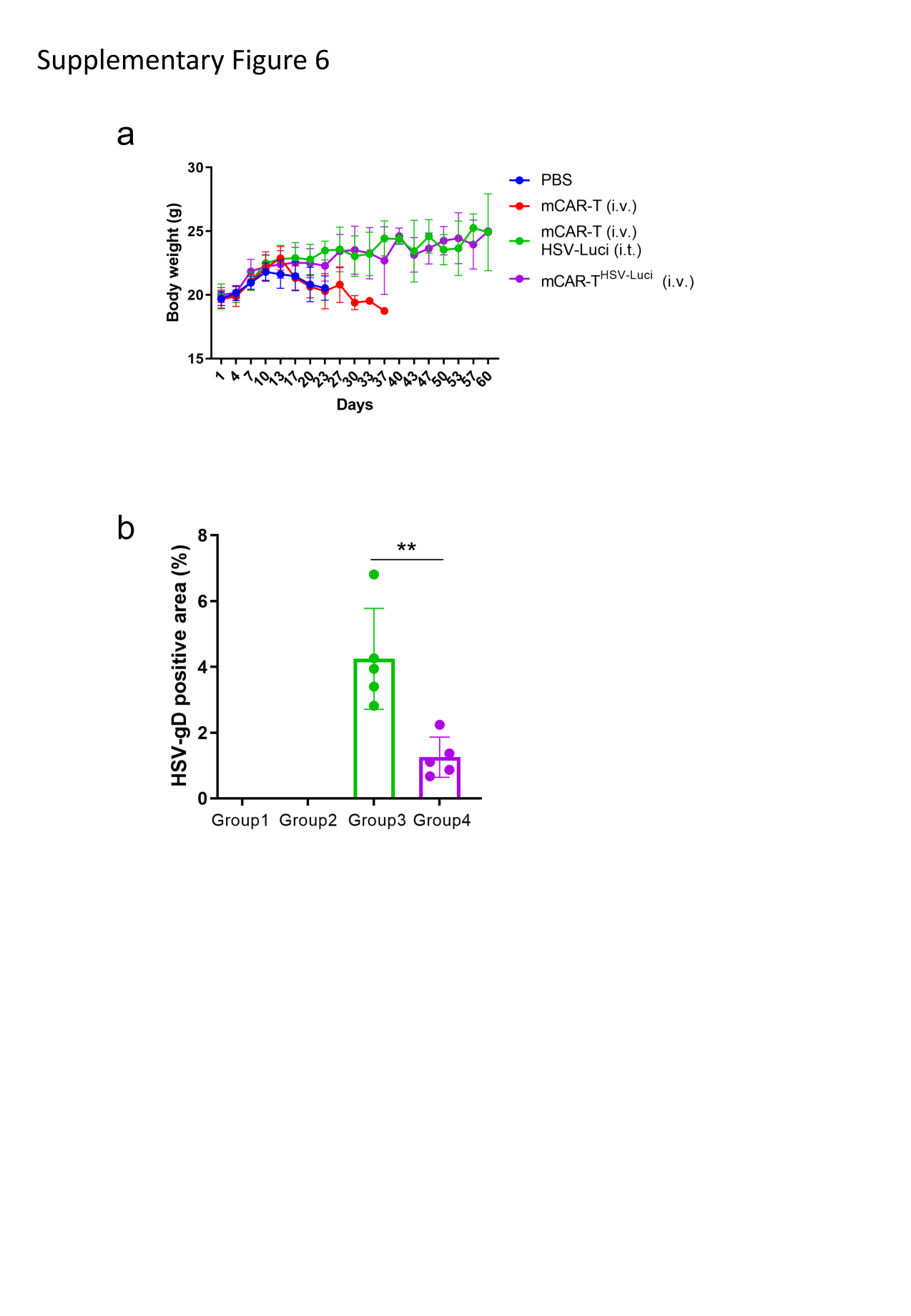
*

**Figure S6** (a) The body weight of each mouse was assessed every three days in the Gl261-hB7-H3 tumor-bearing murine model. n = 10 mice per group. (b) A comparative analysis was conducted to evaluate the efficacy of HSV infection between groups 3 and 4 (n = 5). Data are presented as mean values ± SD and were analyzed by Student’s t test. **P < 0.01. Group 1 received PBS as a control; Group 2 received B7-H3 mCAR-T (i.v.); Group 3 received B7-H3 mCAR-T (i.v.) in combination with HSV-Luci (i.t.); and Group 4 received B7-H3 mCAR-T^HSV-Luci^ (i.v.).

*
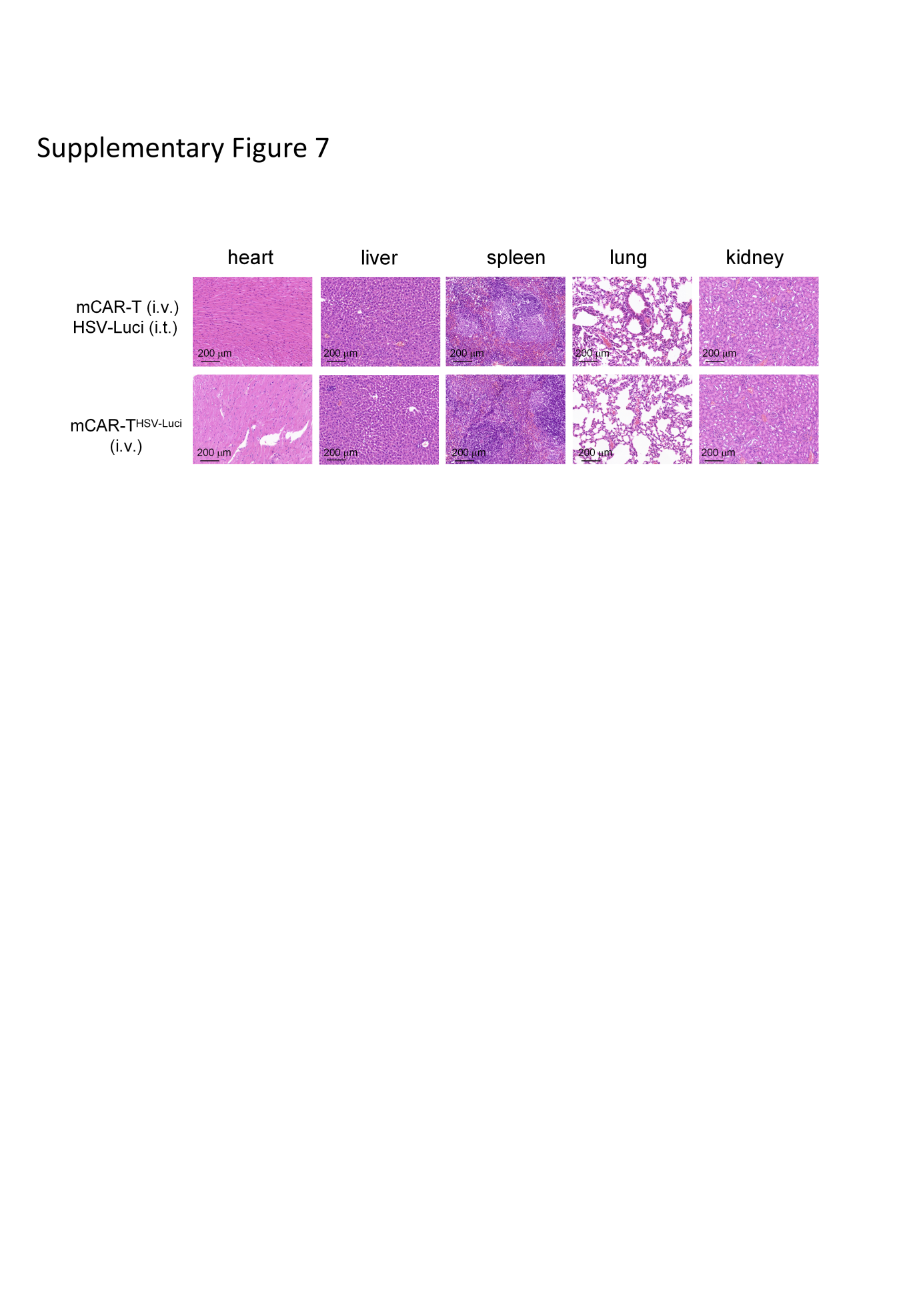
*

**Figure S7** H&E staining was performed on heart, liver, spleen, lung, and kidney specimens following the administration of B7-H3 mCAR-T (i.v.) in combination with HSV-Luci (i.t.) or B7-H3 mCAR-T^HSV-Luci^ (i.v.) treatment. The scale bar is 200 μm.
